# Six years of robots

**DOI:** 10.1155/S146392469100007X

**Published:** 1991

**Authors:** G. F. Plummer, G. Waterworth, W. Roberts

**Affiliations:** Safety of Medicines Department ICI Pharmaceuticals Alderley Park Cheshire Macclesfield SK10 4TG UK


Journal of Automatic Chemistry, Vol. 13, No. 1 (January–February 1991), pp. 29–37

### Six years of robots*

**G. F. Plummer, G. Waterworth and W. Roberts**
*Safety of Medicines Department, ICI Pharmaceuticals, Alderley Park, Macclesfield, Cheshire SK10 4TG, UK*

### Introduction

1984 saw the inception of automated sample preparation for drug analysis at ICI Pharmaceuticals (Macclesfield, UK) in the form of a Zymark (Zymate I) laboratory robot, which was installed in October of that year.

Interest in this form of automation was stimulated by the pioneering work of Eugene Lewis [1], who was active in this technology at ICI Americas.

By July 1985, an automated procedure using liquid/liquid extraction and HPLC analysis for the quantitative measurement of an ICI development drug in plasma had been developed and validated [2].

On purchase of a second robot in 1986 a policy of cloning the systems and developing multitasking analytical robots was adopted, so that all 'drug analysing' robots would be able to perform any subsequently automated method. This approach has led to a high degree of versatility and usage of the individual systems.

Automated procedures now exist that use either single-liquid or solid-phase extractions, or multiple combinations of either or both isolation techniques. These procedures are used to prepare samples for analysis by HPLC, GLC, RIA or ELISA.

Although the major robotic effort has been directed towards drug analysis and the generation of pharmacokinetic data, additional systems have been set up within the Group's Metabolism function and the Departmental Dispensary. The Metabolism robot has been successfully applied to the automation of a Packard 306 Oxidizer, for the measurement of total radioactivity and it is our only PyTechnology system. The Dispensary robot was set up to automate the addition of sterile water to septum-sealed medical vials containing an antibiotic material.

### Multitasking robots

The policy of developing multitasking analytical robots came about from the desire to maximize the potential that the Zymate robots offered to our analytical needs. In 1984/1985, liquid extractions were the foundation of our analytical methods, and automated solid-phase sample preparation in the form of the AASP (Analytichem International, Harbor City, California, USA) was not readily accepted because the apparatus was unreliable. However, the use of Bondelut cartridges was becoming increasingly popular and was the obvious next target for robotic automation.

At this point, the decision was made to develop robotic systems, capable of executing any of our liquid- or solid-phase extraction methods. Also, single-function systems were considered to be unproductive, since there would be times when a particular assay type would not be required, resulting in idle robots. Therefore the second robot system incorporated solid-phase capability, in addition to liquid extraction by vortex.

The main drawback of these Zymate systems was that, although vortex extraction is fast, efficient, reliable and easy to use, the number of organic solvents that vortex well with aqueous solutions is limited.

The efficiency of liquid/liquid extractions is dependent on the effective mixing of the liquid layers to promote a rapid and quantitative recovery of analytes. Previous experience with linear shakers had shown that unless vigorous agitation was used, this form of extraction was slow and prone to poor recoveries. It was for this reason that the Zymark linear shaker – which oscillates gently – was considered unsuitable for our use.

Tumble mixers were popular in our laboratory: they had demonstrated efficient mixing of liquid layers and were known to give efficient and reliable recoveries. The need for such a device, suitable for use on the Zymate robots, led to the development of a rotary mixer. Implementation of this device allowed extractions using organic solvents, which were unsuitable for vortexing, to be automated. Even though these devices worked well, assays using this technique were dogged by the necessity to screw-cap extraction tubes, which is a far from reliable process on a Zymate robot. Consequently, they have now been replaced by Zymark tumble mixers, where screw-capping is not required.

As the demand for solid-phase extraction increased, the use of different size cartridges adversely affected the versatility of the robots, as only one size of cartridge could be accommodated on any single table. Hence certain assays could only be catered for on specific machines. This problem was resolved by designing a multifunctional Bondelut workstation capable of accepting any size cartridge (see the section on Special modules).

The robot systems at ICI Pharmaceuticals are capable of performing liquid extractions by vortex or tumble mixing, along with solid-phase extractions on any type of Bondelut cartridge. Combinations of these techniques are also possible, thus making the systems truly versatile and multitasking.

* Abstract published in *Journal of Automatic Chemistry*, Vol. 12, No. 6.

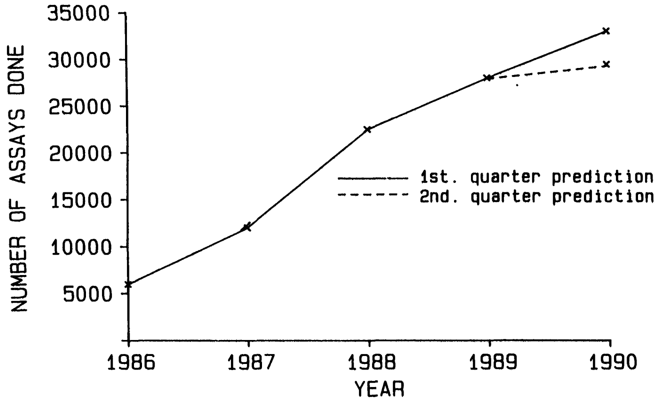

*Figure 1. Drug assays: annual sample throughput.*

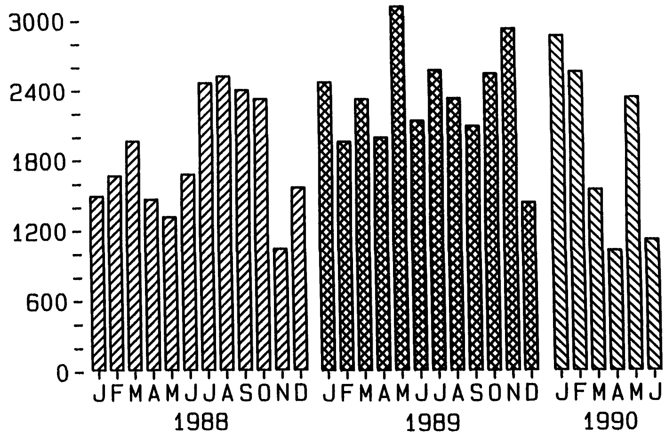

*Figure 2. Total assays per month.*

*Table 1. Robot usage 1988 and 1989.*

| *1988* | % Available time | Runs done | Total samples | Average per run |
|---|---|---|---|---|
| Robot 1 | 71 | 171 | 10 758 | 63 |
| Robot 2 | 71 | 160 | 7704 | 48 |
| Robot 3* | 67 | 73 | 3491 | 38 |
| *1989* | | | | |
| Robot 1 | 65 | 157 | 10 874 | 69 |
| Robot 2 | 63 | 151 | 7847 | 52 |
| Robot 3 | 64 | 140 | 8491 | 61 |

* Since August 1988.

### System usage and throughput

The first routine method at ICI went live in 1985. Since then, this form of automated sample preparation has been used for the analysis of 15 ICI development compounds, with two further analytical methods for one particular compound. In 1986 around 5500 assays were performed robotically, this figure increased to about 12 000 in 1987 and 22 500 in 1988. By 1989, three robotic systems were processing 28 000 assays annually. Initially, figures for 1990 indicated that the sample throughput would exceed 33 000 assays; however, due to the unfortunate demise of two development projects, the 1990 throughput will probably only slightly exceed that of 1989 (figure 1).

The percentage of time available (days) that the robot systems were actually used in 1988 and 1989 are shown in table 1. These data take into account national holidays and unavailability due to programming, upgrading or servicing. Breakdowns do not adversely affect the time available for use, since, in the majority of cases, systems are repaired and are usable on the same day. The average usage for all the systems was 70% for 1988 and 64% for 1989, i.e. 6–7 days per fortnight. Table 1 also shows that the average number of assays per run (all robots) was 53 and 61 for 1988 and 1989, respectively. Although these data show that the systems are used between 64 and 70% of the time, analysing around 50 to 60 samples per run, the actual throughput per month varies considerably (figure 2). Throughputs can vary from just over 3000 per month (May 1989) down to 1100 per month (April 1990).

### Robot performance

Performance of the Zymate robot systems will be discussed as two topics.

(1) The effect on robotic sample preparation time due to system upgrades.

(2) The assessment of system performance with respect to the quality of analytical data.

### *Robotic sample preparation time*

Sample preparations involving single forward liquid/liquid extractions have been in use on our Zymate systems since 1985 and a typical procedure is outlined below.

(1) To a pre-aliquoted biological sample add the internal standard, buffer and extraction solvent.

(2) Vortex the mixture to promote extraction.

(3) Centrifuge to separate the layers.

(4) Remove an aliquot of the upper organic layer and transfer to a clean tube.

(5) Evaporate to dryness under nitrogen.

(6) Add HPLC solvent to the dry residue.

(7) Vortex to dissolve the residue.

(8) Inject an aliquot onto the chromatograph.

In 1985, samples were prepared in groups of six up to the evaporation stage, and then they were placed into an evaporation module. Robot hand changes were then required to move an evaporation manifold over the set of tubes. Once dry, these tubes would be removed from the evaporation module to be replaced by the next set of prepared samples. Owing to the hand changes and manifold manipulations, the robotic time for sample preparation was 10 min.

*Table 2. Sample preparation times. Liquid/liquid extraction – vortex.*

| 1985 | 1986 | 1988 | 1990 |
|---|---|---|---|
| 10 min | 7·5 min<br>Evaporator: pneumatic actuator<br>Serialization | 6·5 min<br>Zymate II<br>CPU 3<br>Tactile sense<br>Speed ×2<br>Transition positions | 5·6 min<br>Processor speed<br>More error detection |

In 1986, a pneumatic actuator was added to the evaporation module, thus removing the necessity for hand changes. Additionally, the sample preparation sequence was serialized. This resulted in a decrease in robotic time to 7·5 min.

In 1988, a third Zymate robot was installed, and the two older systems were upgraded to Zymate II. The Zymate II improvements, which included CPU3, tactile sense, speed ×2 and transition positions, reduced the robotic time to 6·5 min. The rate-limiting step for sample throughput was no longer attributable to the robotic time and was now chromatographically limited.

A fourth Zymate system, incorporating the System 5 controller, was installed in July 1989, and the three other robots were upgraded to this standard in May 1990. These improvements reduced the robotic time to around 5·3 min. However, more error detection and grip confirmation routines have been added making the final robotic time 5·6 min (see table 2).

It would appear that the original preparation time of 10 min per sample would still be acceptable according to the average daily throughput of between 50 and 60 samples. However, what is not apparent is that the faster sample preparation times now available are needed for periods of intense workload. Also, the capability to work faster has allowed for more complex procedures (see below), to be automated.

(*a*) To 0·5 ml pre-aliquoted plasma sample, add 1 ml phosphate buffer (pH 7) and 5 ml methyl *t*-butyl ether.

(*b*) Tumble-mix the mixture to extract the analyte.

(*c*) Centrifuge to separate the layers.

(*d*) Remove 4 ml of the organic layer and transfer to a clean tube.

(*e*) Evaporate to dryness under nitrogen.

(*f*) Redissolve the residue into 2·5 ml aqueous methanol (70% methanol/30% water).

(*g*) Add 2·5 ml hexane and vortex mix.

(*h*) Centrifuge to separate the layers.

(*i*) Aspirate off the top layer (hexane) and transfer 2 ml of the lower layer to a clean tube.

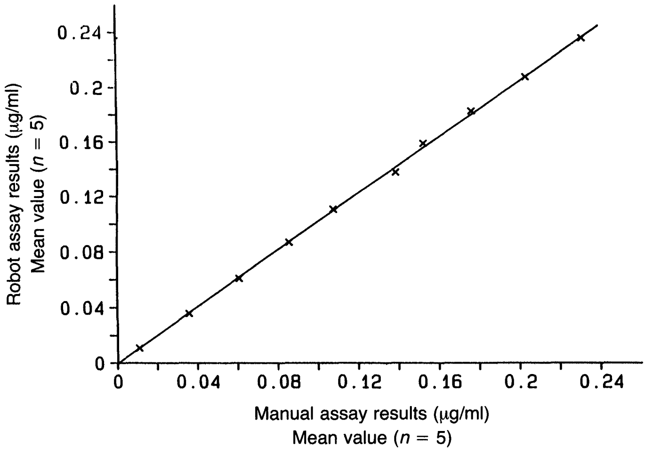

*Figure 3. Manual versus robot performance (Propofol assay).*

(*j*) Add 700 µl chloroform to the aqueous methanol (forming a monophase) and vortex for 20 s.

(*k*) Add a further 700 µl of chloroform and an additional 660 µl of water (now forming two phases) and vortex for 1 min.

(*l*) Centrifuge to separate the layers.

(*m*) Aspirate off the upper phase and add 200 µl of methanol to the lower phase.

(*n*) Evaporate to dryness under nitrogen.

(*o*) Add HPLC solvent and vortex to dissolve the residue.

(*p*) Inject an aliquot onto an enantiomer selective HPLC column.

The above method is the most complex presently in use on the robotic systems, which solely use liquid/liquid extractions/partitions. Serialization is accomplished by using nine sub- (preparation) programs within the top-level program, and complex control of the three centrifugation steps throughout the ramp-up, equilibrium and ramp-down stages of the procedure. At equilibrium, each sample requires three tubes for extractions and nine samples are at various stages of preparation. The robotic time for this method is 9·6 min when using a System 5 controller. It is estimated that a robotic time of between 17 and 19 min would be obtained if performed on a non-System 5 Zymate II robot. Finally, such a method could not have been considered on a Zymate I – apart from taking around 25–30 min per sample – because the software needed exceeds the old Zymate I capacity.

*Quality of analytical data*

Previous papers exhibit numerous examples of accuracy and precision of robotic pipetting, weighing, solvent dispensing, etc., and excellent correlations between manual and robot generated data, often without statistically significant differences. Examples of manual versus robot generated data are shown in figures 3 and 4. The inter- and intra-assay variance of a typical robotic procedure are shown in tables 3 and 4. Typically, these data are generated over relatively short periods of time

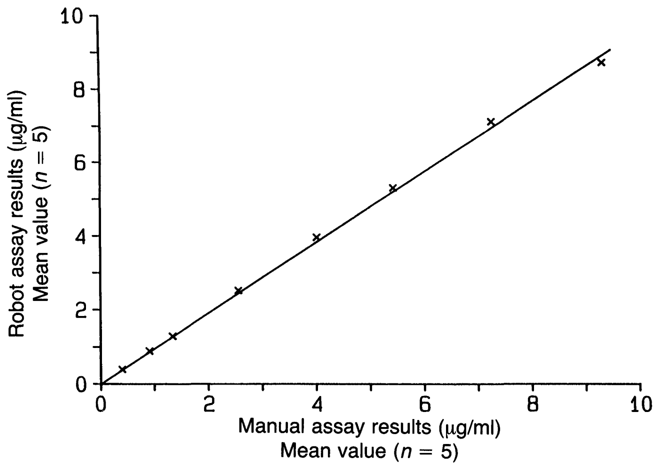

*Figure 4. Manual versus performance (Propofol assay).*

*Table 3. Comparison between the within-assay variation of the manual and robotic analytical methods for Propofol.*

| Manual assay | | Robot assay | |
|---|---|---|---|
| Mean value µg/ml | CV % | Mean value µg/ml | CV % |
| 0·011 | 5·5 | 0·011 | 4·2 |
| 0·036 | 2·2 | 0·036 | 0·8 |
| 0·061 | 2·0 | 0·061 | 0·5 |
| 0·086 | 1·3 | 0·087 | 0·6 |
| 0·108 | 0·5 | 0·111 | 1·3 |
| 0·139 | 2·8 | 0·138 | 0·6 |
| 0·153 | 1·0 | 0·159 | 0·8 |
| 0·177 | 1·0 | 0·183 | 0·4 |
| 0·204 | 2·0 | 0·208 | 1·1 |
| 0·232 | 0·6 | 0·236 | 0·8 |
| 0·40 | 1·8 | 0·40 | 3·8 |
| 0·91 | 2·3 | 0·90 | 1·3 |
| 1·34 | 2·3 | 1·29 | 1·4 |
| 2·56 | 2·4 | 2·52 | 0·7 |
| 4·02 | 3·0 | 3·97 | 0·8 |
| 5·44 | 0·8 | 5·32 | 1·3 |
| 7·27 | 2·4 | 7·14 | 2·0 |
| 9·33 | 2·5 | 8·76 | 1·8 |

using small numbers of samples, although there are a few isolated examples of a large number of comparative assays. Table 4 shows the data generated by the original Zymate system (Robot 1) in 1985 and 1990 for the intra-assay variance of a liquid/liquid extraction procedure with HPLC analysis for compound A. The data illustrate that even after 5 years of use (over 45 000 sample analyses) Robot 1 is still capable of producing quality data. Also, Robot 2, executing the same procedure, furnishes data similar to that of Robot 1.

Invariably, once a new analytical method has been shown to satisfy the required validation criteria, be it either manual or robotic, the procedure is used *ad infinitum.* Providing that the method 'works', i.e. it does not produce erroneous data, validation is generally never revisited. Instrumentation, such as spectrophotometers or balances, can easily be tested for performance to a required specification by using standard solutions or weights. Analytical procedures incorporate numerous quantitative steps which all contribute to the quality of the whole method and it is impractical to periodically re-evaluate these individual steps. Obviously this requires some form of assessment criteria against which analytical methods can be appraised.

*Table 4. Between-assay variation of the robotic analytical method for Propofol.*

| Mean concentration µg/ml (*n* = 5) | CV |
|---|---|
| 0·011 | 4·9% |
| 0·029 | 3·6% |
| 0·066 | 3·5% |
| 0·10 | 3·5% |
| 0·13 | 2·1% |
| 0·18 | 2·9% |
| 0·22 | 4·4% |
| 0·59 | 3·3% |
| 1·07 | 2·4% |
| 1·49 | 1·5% |
| 2·02 | 1·7% |
| 2·62 | 2·2% |

To determine the drug content in biological samples (e.g. blood and plasma) of unknown concentrations, samples are assayed against a series of standards, prepared by adding known amounts of the test compound to aliquots of control biological matrix. Primary solutions of the test compound, and (if used) internal standard, are checked by UV to ensure correct preparation. Sample and standards are then taken through the analytical method, eventually measuring chromatographic peak height(s) or radioactive counts (RIA). For chromatographic assays, standards are fitted by linear regression ($y = mx + c$), whereas RIA or ELISA data are fitted to a four-parameter logistic fit:

$$y = d + \left[ \frac{(a-d)}{1 + \left(\frac{x}{c}\right)^b} \right]$$

where $a$ = maximum binding; $b$ = slope at the ED50; $c$ = ED50; $d$ = non-specific binding; $x$ = concentration of standard; and $y$ = response (cpm for RIA, optical density for ELISA).

The concentration of the 'unknowns' are then calculated using the derived parameters. Samples with concentration values outside the standards range are reassayed with an appropriate standard series.

Assay performance is assessed in two ways: (1) by the 'goodness' of fit of the standards to the algorithm used, i.e. for linear regression an $r$ value tending to 1 and an intercept tending to zero; and (2) the replication of sample analysis. To satisfy the latter requirement, 20% of the samples from an analytical run are reassayed. Providing that the data obtained for the repeat assays

*Table 5. Robot performance. Intra-assay – compound A.*

| Robot 1 | | | | Robot 2 | |
|---|---|---|---|---|---|
| May 1985 | | May 1990 | | May 1990 | |
| µg/ml | (% CV) | µg/ml | (% CV) | µg/ml | (% CV) |
| 0·98 | (3·1) | 1·04 | (1·7) | 1·00 | (3·9) |
| 1·50 | (2·7) | 1·52 | (2·5) | 1·54 | (4·1) |
| 1·96 | (1·5) | 2·05 | (1·3) | 1·96 | (2·1) |
| 4·08 | (3·2) | 4·15 | (1·1) | 4·09 | (3·2) |
| 8·36 | (1·7) | 8·27 | (1·0) | 8·13 | (3·4) |

*Table 6. Replication of assays* (n = *2*)*. Liquid extraction assay – Propofol.*

| Robot | Manual |
|---|---|
| October 1988<br>Average CV = 4·2%<br>(*n* = 31) | September 1988<br>Average CV = 5·5%<br>(*n* = 21) |
| June 1989<br>Average CV = 5·1%<br>(*n* = 16) | June 1989<br>Average CV = 6·5%<br>(*n* = 38) |

agrees to within ±15% of the initial result, the entire batch is accepted, otherwise all the samples would be reanalysed. Medians are calculated for replicates and single assays are taken as found.

Until recently the same criteria have been applied to the robotic assay procedures and the data below are typical of that generated by automated assay methods. Presently under evaluation is the use of quality-control samples to monitor assay performance, and these will eventually replace replicate analyses. It will be interesting at some later date to compare the quality-control data between manual and robotic methods.

In order to assess the variation in assay replication on the robot systems over an extended period of time, data were extracted from study files for two compounds and the average coefficients of variations (CV) were calculated. Different robots were used for each compound and the data relate to that generated on each robot only. This in itself proved difficult to obtain as users rotate from machine to machine. The methods chosen were that for Propofol [3], which is a liquid/liquid extraction method with HPLC fluorescence, using an internal standard (table 6), and a solid-phase HPLC/UV procedure without internal standard, for a carbopenem antibiotic – compound c (table 7). The robotic Propofol data is also compared with that from manual assays carried out at around the same time. The data show that the average CV for both robotic procedures is quite consistent over the periods examined, and the data for the automated Propofol assay indicate a marginally better average CV than that for the manual method.

Paramount to all methods using internal standards is the precision of the volume of internal standard solution

*Table 7. Replication of assays* (n = *2*)*. Solid phase assay – compound c.*

| |
|---|
| March 1988<br>Concentration 0·4 to 36·0 µg/ml<br>Average CV = 4·3%<br>(*n* = 13) |
| June 1988<br>Concentration 0·6 to 63·0 µg/ml<br>Average CV = 4·6%<br>(*n* = 18) |
| March 1988<br>Concentration 0·6 to 34·0 µg/ml<br>Average CV = 4·7%<br>(*n* = 18) |
| June 1988<br>Concentration 0·5 to 43·0 µg/ml<br>Average CV = 4·5%<br>(*n* = 13) |

*Table 8. Internal standard aliquoting (Master Laboratory Station).*

| | Robot 1<br>Syringe size<br>100 µl | Robot 2<br>Syringe size<br>100 µl | Robot 3<br>Syringe size<br>100 µl |
|---|---|---|---|
| 1985 | CV = 0.78% | | |
| 1986 | CV = 0·60% | CV = 0·77% | |
| 1987 | | | |
| 1988 | CV = 0·88% | | CV = 1·04% |
| 1989 | | CV = 0·52% | CV = 0·90% |
| 1990 | CV = 0·73% | CV = 0·48% | CV = 0·94% |

added. This parameter is periodically tested and the results are summarized in table 8. These data exhibit an excellent consistency in the level of precision for all robot systems.

An additional measure, which in most cases can be used as a check on assay performance, is the variability of the internal standard peak height. This generally gives a good indication of the assay performance over the whole procedure, rather than just internal standard addition alone. The CV of the peak heights obtained during the analysis of Propofol samples is given in table 9. The CV is shown to vary from 1·8 to 3·4% when using Robot 2, and from 1·7 to 4·7% for Robot 3. These values are considered very good for this type of assay.

The accuracy and precision of dispensing solvents by Master Laboratory Stations (MLS) is a popular subject of presentations and such data are included in this paper (see the section on the Dispensary robot). As most liquid extraction methods use internal standards, small variations in solvent volumes would not critically affect the analytical procedure. When using procedures which exclude internal standards, the accuracy of solvent addition is checked. It is sufficient to state that volumes are always accurate and precise. It is more interesting to note that, in 1985, an MLS on Robot 1 was tested for dispensing 6 ml volumes of organic solvent and found to

*Table 9. Internal standard peak height variation. Propofol assay.*

| Month/year | CV (%) |
|---|---|
| May 1987 | 2·3 |
| June 1987 | 2·0 |
| October 1987 | 2·1 |
| December 1987 | 3·7 Robot 2 |
| February 1988 | 2·2 |
| March 1988 | 1·8 |
| May 1988 | 3·4 |
| August 1988 | 3·9 |
| September 1988 | 3·5 |
| October 1988 | 2·1 |
| January 1989 | 2·7 Robot 3 |
| March 1989 | 2·0 |
| April 1989 | 1·7 |
| May 1989 | 4·7 |
| June 1989 | 3·2 |

*Table 10. Robot systems – module repairs.*

| | Total modules (number of repairs) | | | | |
|---|---|---|---|---|---|
| | 1986 | 1987 | 1988 | 1989 | 1990 |
| Controller | 2(0) | 2(0) | 3(2) | 4(0) | 4(0) |
| Capper | 2(0) | 2(1) | 3(0) | 4(1) | 4(0) |
| Centrifuge | 2(0) | 2(1) | 3(1) | 4(0) | 4(0) |
| Evaporator | 2(0) | 2(0) | 3(0) | 4(0) | 4(0) |
| Hands | 3(0) | 3(0) | 6(0) | 9(2) | 9(2) |
| MLS | 5(2) | 5(2) | 8(2) | 12(1) | 14(0) |
| PEC | 4(0) | 4(1) | 6(1) | 8(1) | 8(0) |
| Printer | 2(0) | 2(1) | 3(0) | 4(1) | 4(0) |
| Robot | 2(1) | 2(3) | 3(4) | 4(2) | 4(1) |
| Tumbler | | | | 3(1) | 3(0) |
| Vortex | 4(0) | 4(1) | 6(0) | 8(1) | 8(0) |
| Robot 1 | 2 | 7 | 5 | 5 | 1 |
| Robot 2 | 1 | 3 | 3 | 4 | 0 |
| Robot 3 | | | 2 | 1 | 1 |
| Robot 4 | | | | | 1 |
| Zymark | 2 | 8 | 8 | 8 | 1 |
| In-house | 1 | 2 | 2 | 2 | 2 |

give 5·98 ml at 0·05% CV ($n$ = 20). In 1990, the same MLS pipetted 6·0 ml at a CV of 0·12% ($n$ = 20). This robot has performed in excess of 45 000 assays, and the syringe drive on this particular MLS, which is still in its original condition, has been operated over 630 000 times.

### Robot system repairs

Summarized in table 10 are the repairs carried out on the Zymate systems modules since 1986. These are breakdown situations which render the modules unusable. These data indicate the total number of each type of module, and the number of repairs to each module type is given in parentheses. Also detailed is the number of repairs to each individual robot system and the repairs done either by Zymark or in-house. Not included in these data is an annual service carried out at the beginning of September. These data are displayed separately in table 11.

*Table 11. September 1990 Service.*

| | |
|---|---|
| Robot 1 | Vertical motor replacement. |
| Robot 2 | Vertical potentiometer replacement. |
| Robot 3 | Vertical motor replacement. Robot arm bearing adjustment. |
| Robot 4 | Nothing required. |

These data have been collated to indicate the mechanical reliability of the Zymate robots and do not necessarily relate to the overall working reliability of the systems, i.e. the ability to complete analytical runs faultlessly. There are numerous reasons why the robots 'stop working', all of which occur rarely – these are usually easily dealt with and are not identifiable as a systematic error or module problem. However, the summation of these rarities can become significant. Experience with the Zymark robots shows that periods of inexplicable 'instability' occur on the systems, i.e. overnight runs fail to complete. The reasons for these failures are usually different each time and often do not happen again. Runs are normally restarted the next morning and regularly go to completion. These periods of 'instability' can last several weeks before tranquillity returns. As yet, no real reason has been found for these frustrating events.

For a robot system to work reliably, many functions must be executed without fault and analysis of robotic procedures can yield some interesting statistics. Take, for example, the standard liquid extraction procedure carried out on our Zymate robots. For a single sample to be processed, the following activities must take place.

616 commands are issued consisting of:

- 72 program commands;
- 24 maths expressions;
- 179 Easylab commands (if-then, goto, set timer, etc.);
- 341 module commands or module command variables.

In 1989, Robot 1 completed to 10 874 analyses (all liquid extractions) which converts to *6·7 million* commands executed.

A single sample also generates 120 robotic movements (141 including grip) at equilibrium. Therefore, for 1989, a total of *1·30 million* (1·53 million with grip) robotic movements were performed by the robot. Also, to process a single sample, 6·3 m of linear movement by the arm is required, totalling 68 500 m (43 miles) for 1989. These figures are an illustration of the demands placed on the robotic sytems.

### Special modules

Special module development has been a feature of robotic evolution in ICI Pharmaceuticals for the past 5 years and came about from the need to perform particular functions of analytical chemistry for which no Zymark module existed. In the earlier years, Zymark UK did not have the capacity or experience to design and manufacture custom modules, and therefore development times were extended since all customization was carried out at Hopkinton. The desire to expedite new applications in robotics resulted in our own Engineering Research Laboratories being used for module design and manufacture. Consequently, we now have a skilled and experienced facility to draw upon, which permits special modules to be produced in a timely manner. Examples of special engineering developments are the liquid chromatographic injector and the rotary mixer; recent developments include an emulsion detector and a multifunctional Bondelut workstation. Other examples of engineering developments will be described later under the heading 'Dispensary robot' and the automation of the Packard 306 Oxidizer.

Inherent to liquid/liquid extraction procedures is the potential for emulsion formation, particularly when using hydrocarbon solvents. These are often difficult to break by centrifugation alone. The necessity to detect these emulsions became essential for the automation of an analytical method involving blood and cyclohexane. The variability of emulsion formation was such that the volume of solvent incorporated in the emulsion could range from all to nothing. Also, which samples were likely to emulsify was unpredictable, therefore vital to the automation of this method was the detection of these emulsions. The problem was resolved by the use of a fibre-optic, through beam, infra-red detector. This device operates on the principle that a blood/solvent emulsion absorbs the infra-red light, thus breaking the beam, whereas glass and clear solvent permits the light to pass through. By using such a device, these types of emulsions are easily detectable. Also, by viewing the extract mixture, initially at just above the expected blood/solvent interface position and then if necessary at higher positions, equivalent to 1 ml volumes of solvent, decisions can be made as to the volume of solvent removable from the mixture, within set limits. Sample extracts failing the emulsion test can be aborted at this stage of the assay procedure.

Solid-phase sample preparation has been in use on the robotic systems since 1986, with two styles of workstation required to use the 1 ml or 10 ml (LRC) cartridges. Owing to space constraints, these are on separate robotic worktables. An upsurge in the use of these isolation procedures, along with the philosophy of developing multitasking robots, has promoted the production of a multifunctional Bondelut workstation (figures 5 and 6). The carousel design to hold cartridges, along with the stepped pyramid-shaped sealing head, allows the workstation to accept all sizes of Bondelut cartridge. This device now permits any solid-phase method to be carried out on any robotic system, regardless of cartridge size.

### Dispensary robot

A Zymark robot has been set up in the Departmental Dispensary to prepare injectable formulations of an antibiotic material in septum-sealed medical vials. This

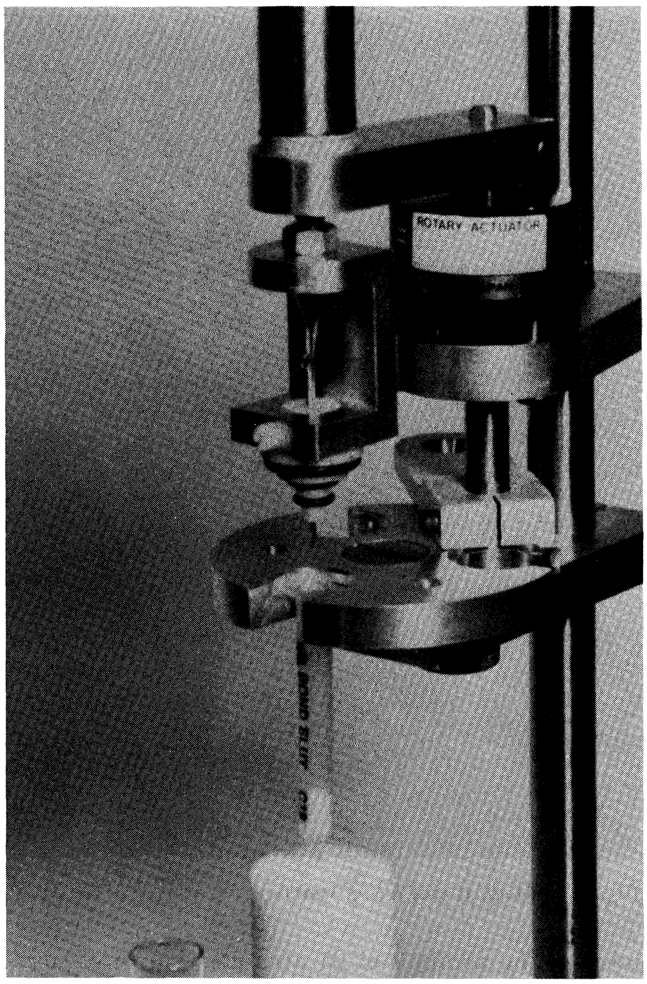

*Figure 5. Bondelut multistation.*

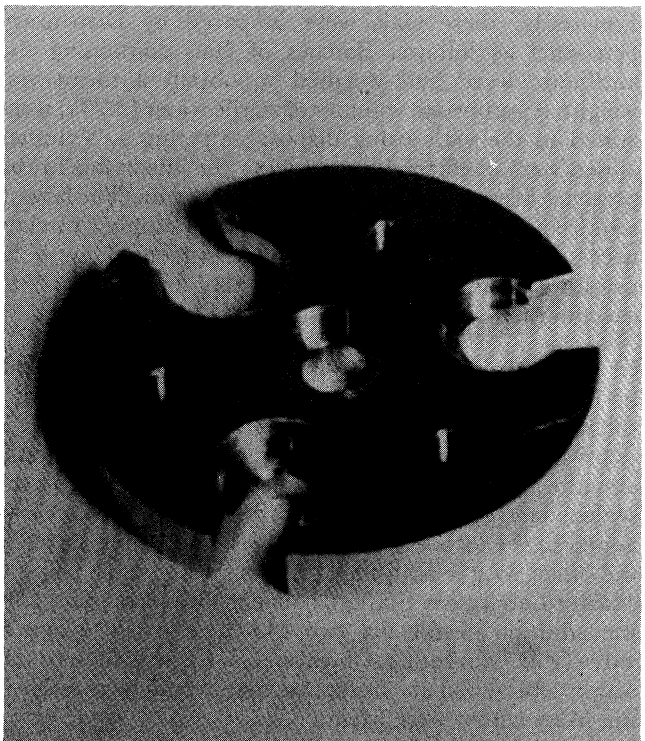

*Figure 6. Bondelut multistation carousel.*

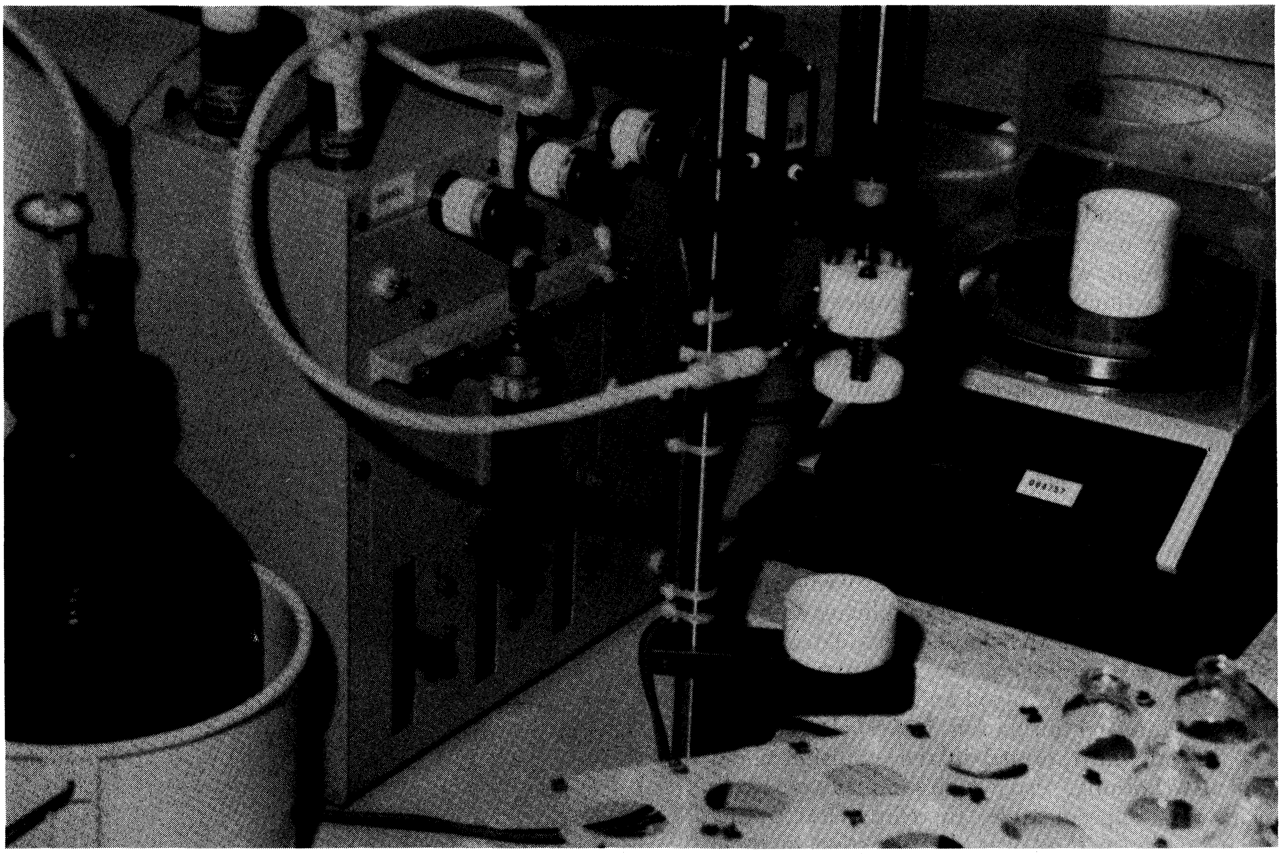

*Figure 7. Medical vial filling station.*

involves the addition of an accurate volume of sterile water to the vials and subsequent mixing to ensure dissolution.

Previously, these vials were prepared by Dispensary personnel as follows. Batches of vials containing the antibiotic were bulk-weighed to obtain a mean vial weight. Appropriate volumes of sterile water (±2%) were added to the vials using disposable syringes. Volumes added were confirmed by weighing the filled vials in the presence of an additional member of the team. The labour requirements for the preparation of the number of vials necessary for the studies was five personnel for 4 h, resulting in an extended working day for these staff to execute their normal duties.

In order to automate this process and supply the necessary number of prepared vials, a single Zymark robot was commissioned. A workstation, incorporating a twin needle arrangement attached to a pneumatic actuator, was specifically designed to pierce the septum-sealed vials (figure 7). Using this device, sterile water was added to the vials via one needle whilst venting through the other. Water additions (22·9 ml) were done using a Master Laboratory Station, connected to a reservoir and the addition needle via two Bio-chem two-way pinch valve (P.D. Marketing, Chichester, UK); this allowed the water to be added at a faster rate than would be possible if the MLS valves were used.

Prior to the installation of this system, trials indicated that in order to dissolve completely the antibiotic

*Table 12. Water addition – sealed medical vials.*

| | Day 1 | Day 21 | Day 41 |
|---|---|---|---|
| Number done | 32 | 56 | 58 |
| Mean volume | 22·97 | 22·93 | 22·92 |
| SD | 0·015 | 0·06 | 0·007 |
| CV | 0·063% | 0·026% | 0·028% |
| Accuracy | 100·3% | 100·1% | 100·1% |
| | Day 61 | Day 81 | Day 101 |
| Number done | 58 | 59 | 48 |
| Mean volume | 22·91 | 22·88 | 22·89 |
| SD | 0·005 | 0·039 | 0·010 |
| CV | 0·024% | 0·17% | 0·042% |
| Accuracy | 100% | 99·9% | 100% |
| | Day 121 | Day 141 | Day 161 |
| Number done | 59 | 60 | 60 |
| Mean volume | 22·90 | 22·90 | 22·84 |
| SD | 0·009 | 0·016 | 0·009 |
| CV | 0·039% | 0·068% | 0·039% |
| Accuracy | 100% | 100% | 99·7% |

material a vortexing time of about 5 min was necessary. Since the robotic time was just under 2 min, serialization required the use of four vortexers, three of which would be active at any one time. The cumulative effect of these active vortexers produced severe vibration of the robotic worktable. This eventually caused connectors in the robot wrist-box to work loose, thus disabling certain functions of the robot hand (particularly the grip), resulting in 'crashes'. The solution was to mount the vortexers on anti-vibration plates, which were constructed from four Metalastik, Instrumountings (W. Christie and Grey Ltd, Tonbridge, UK) sandwiched between two 0·375 in polypropylene plates. After doing this, no vibration was transmitted to the worktable.

Apart from the vastly reduced labour requirements, in terms of technician time to load the robot of basic materials and unload the finished vials, other additional benefits were obtained. Since the robot works singularly on vials, data can be generated as to the tare and gross weights of the vials, and, consequently, the nett weights/volumes added to each individual vial. These data can also be neatly tabulated and recorded. Accuracy and precision of volumes added is a natural consequence of using mechanical dispensers, and validation showed that the accuracy of the water volumes added ranged from 99·9 to 100·5% of that required volume with CVs of between 0·05 and 0·15%. Data generated from routine use of the system are shown in table 12.

It is interesting to note that the manual filling of vials using disposable syringes required frequent needle changes (every five or six vials) to prevent coring and addition of septum debris to the vials. The robotic system, with its custom-designed vial piercing and water addition station, was used 4500 times with no signs of septum coring, although the needles were changed at this point due to the vent needle blocking. Subsequent needle changes were done every 2000 vials.

### Packard Oxidizer automation

The measurement of total radioactivity in biological samples is typically carried out by combustion analysis on Packard Oxidizers. This process is highly manually intensive and requires total commitment of the analyst's time.

The automation of a Packard 306 Oxidizer using a Zymark robot was previously described by Neil *et al.* [4].

The Packard Oxidizer is unquestionably robot unfriendly in its unmodified form and frequently lives up to its reputation of being unreliable. To overcome the problems experienced by the previous automation, modifications have been made to a Packard 306 Oxidizer, combined with additional or improved error-sensing devices.

The major problem, highlighted by Neil *et al.*, is the interface between the Packard Oxidizer and the robot, this being the tilted vial-carriage mechanism. Access is restricted by the sides of the carriage and a central stud, requiring vials to be handled by the screw threads. Also, the square edges of the vial locations are not receptive to flat-bottomed vials. Removal of the carriage sides and the central stud, along with the insertion of robot friendly sleeves into the carriage, removed all vial loading problems.

For error-sensing, the previous automation incorporated a low-pressure/vacuum switch into the Oxidizer exhaust pipe in order to confirm oxygen gas flow. Failure to activate the switch is indicative of leaks, blockages or failure to place a vial into the Oxidizer. Evaluation of such a device showed that, at best, oxygen flow rate reductions of at least 40% were necessary before errors were detectable, representing a 25% reduction in the radioactivity recoverable. This was considered of no real benefit. Oxygen flow rates may, however, be measured (l/min), using a Brooks 5850TR Thermal Mass Flowmeter (Brooks Instrument Division, Emerson Electric UK Ltd, Stockport, UK), connected in line to the Packard exhaust and the A/D of a power and event controller. When using this device, flow reductions of between 8 and 10% can easily be detected.

Additional error sensing has also been incorporated into this system to confirm successful ignition of the samples and correct dispensing of the solvents. The latter is easily confirmed using a balance and weighing the full vials to ±1 g. Successful ignition is confirmed by monitoring the combustion chamber temperature using a thermocouple.

These developments have resulted in a reliable robot/oxidizer system incorporating diagnostics to confirm, oxygen flows, sample ignition and combustion and correct dispensing of solvents.

### Acknowledgements

We would like to make a special reference to the first-class support we have received from the Research Engineering Group at ICI, particularly Ken Gallimore, Steve Johnson, Jim Brennan and Mike Dean.

### References

1. Lewis, E. C., In *Advances in Laboratory Automation – Robotics 1984*, Eds Hawk, G. L. and Strimatis, J. R. (Zymark Corporation, 1984), 237–255.
2. Plummer, G. F., *Advances in Laboratory Automation – Robotics 1986*, Eds Hawk, G. L. and Strimatis, J. R. (Zymark Corporation, 1986), 47–70.
3. Plummer, G. F., *Journal of Chromatography*, **421** (1987), 171–176.
4. Neil, W., Yang, J. J. and Roy, T. A., *Advances in Laboratory Automation – Robotics*, Eds Hawk, G. L. and Strimatis, J. R. (Zymark Corporation, 1988), 69–82.

